# Concomitant surgical ablation for atrial fibrillation in mitral valve surgery: a systematic review of clinical outcomes and surgical strategies

**DOI:** 10.3389/fcvm.2026.1857413

**Published:** 2026-08-13

**Authors:** Zhanar Nurbay, Rustem Tuleutayev, Maxat Zhakayev, Auyeskhan Dzhumabekov, Nurzhan Musrepov

**Affiliations:** 1 Department of Cardiac Surgery, SCE “City Cardiology Center”, Almaty, Kazakhstan; 2 Department of Cardiac Surgery, JSC “Research Institute of Cardiology and Internal Diseases”, Almaty, Kazakhstan; 3 Department of Clinical Work, Kazakh Medical University “KSPH”, Almaty, Kazakhstan

**Keywords:** atrial fibrillation, cardiac surgery, cox-maze IV, cryoablation, mitral valve surgery, radiofrequency ablation, rhythm control, stroke prevention

## Abstract

**Background:**

Atrial fibrillation frequently coexists with mitral valve disease and is associated with adverse clinical outcomes. Concomitant surgical ablation has emerged as an effective strategy to restore sinus rhythm during mitral valve surgery.

**Methods:**

A systematic review was conducted according to PRISMA guidelines. Electronic databases were searched for studies published between January 1991 and January 2026. Eligible studies included adult patients undergoing mitral valve surgery with concomitant surgical ablation. Due to heterogeneity, a qualitative synthesis was performed.

**Results:**

A total of 25 studies were included. Early evidence established the Cox-Maze III procedure as an effective approach with high rates of sinus rhythm restoration. Contemporary data demonstrate that the Cox-Maze IV procedure provides comparable efficacy with reduced technical complexity. Long-term follow-up showed sustained sinus rhythm in a substantial proportion of patients, with possible reductions in thromboembolic events and possible survival benefit reported in some observational studies. However, interpretation of these findings is limited by clinical and methodological heterogeneity. However, an increased risk of permanent pacemaker implantation remains a consistent finding. Procedural success is influenced by lesion set configuration, completeness of ablation, and surgical approach.

**Conclusions:**

Concomitant surgical ablation during mitral valve surgery is consistently associated with better rhythm outcomes, while evidence for harder clinical endpoints, including stroke and survival, remains less certain. Individualized surgical approaches remain essential to balance rhythm efficacy, procedural complexity, and the risk of permanent pacemaker implantation.

## Introduction

Atrial fibrillation is the most common sustained cardiac arrhythmia and remains a major contributor to cardiovascular morbidity and mortality worldwide. It is associated with an increased risk of thromboembolic stroke, heart failure, repeated hospitalizations, reduced exercise tolerance, and impaired quality of life. Common symptoms include palpitations, dyspnea, fatigue, dizziness, and reduced functional capacity, although some patients remain asymptomatic until complications occur. The burden of atrial fibrillation is particularly pronounced in patients with structural heart disease, especially those with mitral valve pathology, in whom chronic pressure or volume overload promotes left atrial enlargement, fibrosis, and electrical remodeling, thereby facilitating the development and persistence of arrhythmia (1).

Mitral valve disease and atrial fibrillation frequently coexist in clinical practice. Previous studies have shown that atrial fibrillation is present in approximately 20%–42% of patients undergoing mitral valve surgery (2, 3). The presence of preoperative atrial fibrillation in this population is associated with worse perioperative and long-term outcomes, including higher risks of stroke, heart failure progression, and mortality. Importantly, mitral valve surgery offers a unique therapeutic opportunity for concomitant rhythm correction because surgical exposure of the left atrium is already required as part of the primary procedure. This creates favorable conditions for performing surgical ablation without the need for separate invasive access.

Over the past two decades, surgical treatment strategies for atrial fibrillation have evolved considerably. The traditional cut-and-sew Cox-Maze III procedure demonstrated excellent long-term efficacy but was technically complex and associated with prolonged operative times. To improve procedural feasibility and facilitate wider adoption, less invasive energy-based lesion sets were developed, leading to the widespread implementation of the Cox-Maze IV procedure using bipolar radiofrequency ablation (RFA), cryoablation, or combined approaches. The Cox-Maze IV procedure is currently considered the standard surgical ablation strategy because it provides high rates of sinus rhythm restoration while reducing technical complexity compared with earlier techniques. Reported freedom from atrial fibrillation and antiarrhythmic drug use at 1 year after Cox-Maze IV reaches approximately 85% and 93%, respectively (4).

The use of concomitant surgical ablation during mitral valve surgery has increased steadily, with registry data showing that its use rose from 52% to 62% over the past decade (5). This trend reflects growing recognition of the potential benefits of rhythm restoration in this high-risk population. Several studies have demonstrated that patients undergoing concomitant atrial fibrillation ablation during mitral valve surgery have improved maintenance of sinus rhythm, reduced thromboembolic events, better symptom control, and potentially improved long-term survival compared with those undergoing mitral valve surgery alone (6, 7). However, important questions remain regarding the optimal lesion set, choice of energy source, extent of atrial ablation, and role of minimally invasive surgical approaches.

Current international clinical practice guidelines recommend concomitant surgical ablation in appropriately selected patients with atrial fibrillation undergoing mitral valve surgery to improve rhythm outcomes and reduce complications (8, 9). Nevertheless, evidence remains heterogeneous because available studies differ in patient characteristics, atrial fibrillation type, lesion patterns, energy modalities, follow-up duration, and rhythm monitoring strategies. In addition, newer minimally invasive techniques and emerging technologies continue to expand therapeutic options, but their comparative effectiveness and long-term safety remain incompletely defined.

Several previous systematic reviews and meta-analyses have evaluated surgical ablation for atrial fibrillation during mitral valve or other concomitant cardiac surgery (10–13). These analyses have established that adding surgical ablation improves postoperative sinus rhythm restoration compared with mitral valve surgery alone. However, most prior reviews primarily focused on pooled rhythm outcomes or comparisons between ablation and no ablation. Consequently, several clinically important sources of heterogeneity remain insufficiently examined, including differences between biatrial and left atrial lesion sets, completeness and transmurality of lesion formation, radiofrequency vs. cryothermal energy delivery, management of the left atrial appendage, and variation in rhythm-monitoring protocols and definitions of treatment success. Moreover, earlier syntheses included limited evidence regarding contemporary Cox-Maze IV techniques, minimally invasive mitral valve surgery, and recently reported long-term outcomes such as stroke, survival, and permanent pacemaker implantation.

The present systematic review was therefore designed to extend previous quantitative syntheses by examining how procedural strategy and methodological variation influence the reported efficacy and safety of concomitant surgical ablation specifically in patients undergoing mitral valve surgery. In addition to summarizing rhythm and clinical outcomes, the review evaluates the evolution from the cut-and-sew Cox-Maze III procedure to contemporary Cox-Maze IV approaches; compares lesion-set configurations and energy modalities; examines left atrial appendage management and its potential contribution to thromboembolic outcomes; assesses the influence of rhythm-monitoring intensity and recurrence definitions; and considers the implications of minimally invasive surgical approaches. By integrating these clinical and methodological dimensions, this review aims to clarify the factors underlying variability across studies and to identify evidence gaps relevant to individualized procedural selection and future comparative research.

## Methods

### 2.1 Study design and reporting framework

This systematic review was conducted in accordance with the 2020 Preferred Reporting Items for Systematic Reviews and Meta-Analyses (PRISMA) statement. The aim of this review was to comprehensively evaluate the efficacy and safety of concomitant surgical ablation for atrial fibrillation in adult patients undergoing mitral valve surgery, with particular focus on lesion sets, energy modalities, rhythm outcomes, perioperative safety, and long-term clinical results. Given the expected heterogeneity in study designs, patient populations, surgical techniques, and follow-up protocols, a qualitative evidence synthesis was planned.

### 2.2 Eligibility criteria

Studies were selected according to predefined eligibility criteria based on the Population, Intervention, Comparator, Outcomes, and Study design (PICOS) framework. Eligible studies included adult patients undergoing mitral valve surgery with preoperative atrial fibrillation who received concomitant surgical ablation using Cox-Maze procedures, radiofrequency ablation, cryoablation, or combined lesion sets. Randomized controlled trials, prospective cohort studies, retrospective cohort studies, and comparative observational studies were considered eligible if they reported at least one clinically relevant outcome, including sinus rhythm restoration or maintenance, recurrence of atrial arrhythmia, stroke, mortality, permanent pacemaker implantation, perioperative complications, or follow-up outcomes. Only full-text articles published in English were included. Case reports, small case series, conference abstracts without full text, editorials, narrative reviews, expert opinions, duplicate publications, studies not specifically evaluating patients undergoing mitral valve surgery, and studies without extractable outcome data were excluded.

### 2.3 Information sources and search strategy

A systematic literature search was performed in PubMed, Embase, Cochrane Library, and Web of Science to identify relevant studies published between January 1991 and January 2026. The search was conducted in January 2026. In addition, the reference lists of included studies and relevant review articles were manually screened to identify further eligible publications. The search strategy combined controlled vocabulary and free-text terms related to atrial fibrillation, mitral valve surgery, and surgical ablation. The main search concepts included atrial fibrillation, mitral valve surgery, mitral valve repair, mitral valve replacement, surgical ablation, Cox-Maze procedure, radiofrequency ablation, cryoablation, Maze procedure, and minimally invasive surgery. A detailed database-specific search strategy is provided in Supplementary Table S1.

### 2.4 Study selection

**Figure 1:**
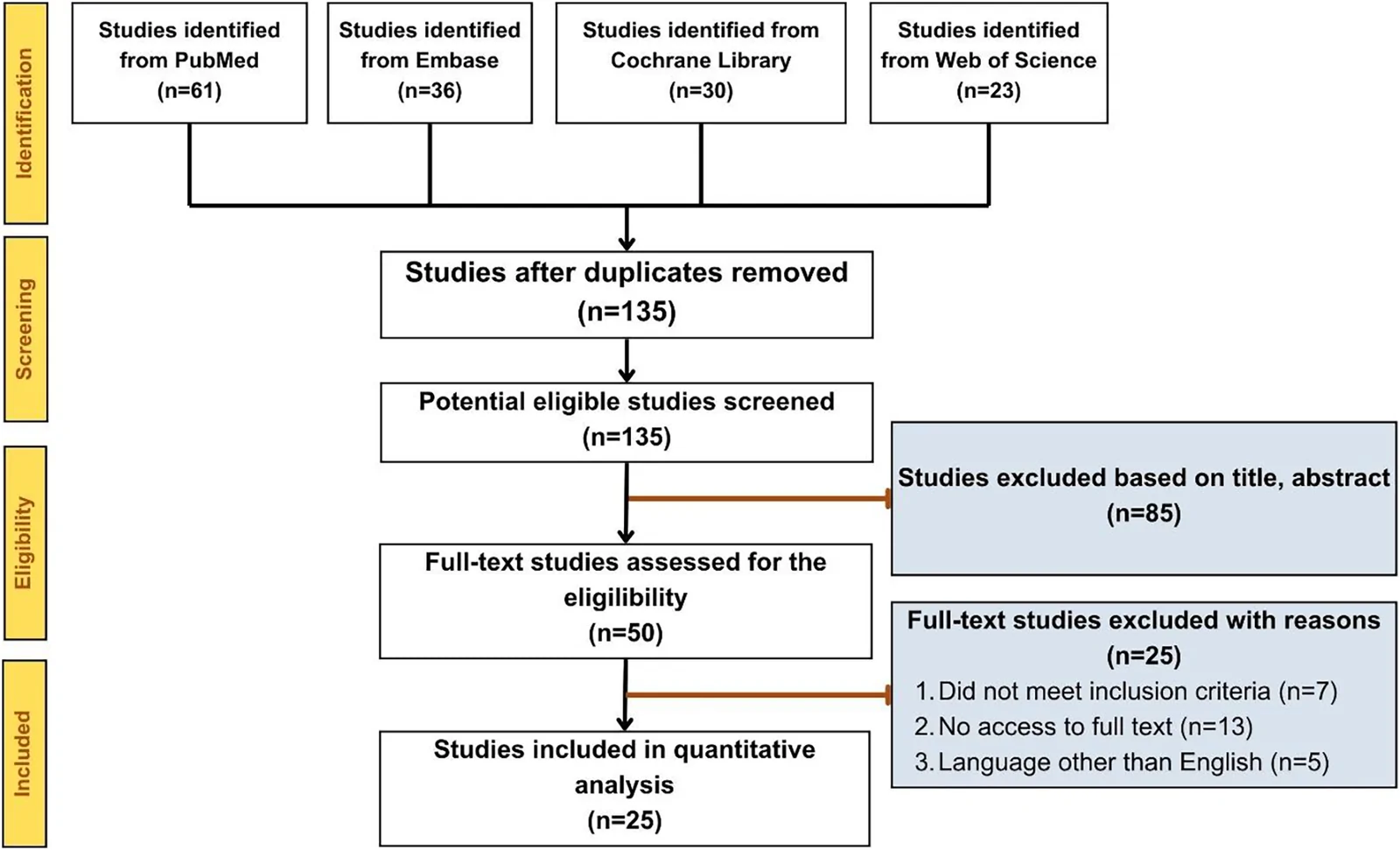
PRISMA flow diagram.

Two reviewers independently screened titles and abstracts identified through the database search. Full texts of potentially eligible studies were subsequently assessed according to the predefined inclusion and exclusion criteria. Disagreements were resolved through discussion and, when necessary, consultation with a third reviewer. The study selection process is presented in the PRISMA flow diagram (Figure 1).

### 2.5 Data extraction and outcomes

Data were extracted independently by two reviewers using a standardized data extraction form. Extracted variables included study characteristics, patient demographics, type and duration of atrial fibrillation, mitral valve pathology, surgical approach, ablation lesion set, energy modality, follow-up duration, and reported clinical outcomes. The primary outcome was maintenance of sinus rhythm during follow-up. Secondary outcomes included recurrence of atrial fibrillation, stroke or thromboembolic events, perioperative and long-term mortality, permanent pacemaker implantation, major postoperative complications, and length of hospital stay, when available. Any discrepancies in extracted data were resolved by consensus.

### 2.6 Risk of bias assessment

The methodological quality of non-randomized studies was assessed using the Risk Of Bias In Non-randomized Studies of Interventions (ROBINS-I) tool. Randomized controlled trials, where applicable, were assessed using the Cochrane Risk of Bias 2 tool. Quality assessment was performed independently by two reviewers, and disagreements were resolved through discussion.

### 2.7 Data synthesis

A qualitative synthesis was performed because of substantial clinical and methodological heterogeneity among the included studies. Differences in patient populations, atrial fibrillation subtype, lesion sets, energy modalities, rhythm monitoring methods, and follow-up duration precluded meaningful quantitative pooling. Therefore, meta-analysis was not considered appropriate. Because no formal meta-analysis was conducted, statistical assessment of publication bias was not performed. A qualitative synthesis was performed because of substantial clinical and methodological heterogeneity among the included studies. Differences in patient populations, atrial fibrillation subtype, lesion sets, energy modalities, left atrial appendage management, rhythm-monitoring methods, and follow-up duration precluded meaningful quantitative pooling. Therefore, meta-analysis was not considered appropriate. Because no formal meta-analysis was conducted, statistical assessment of publication bias was not performed. The findings were interpreted with consideration of study design, risk of bias, consistency across studies, sample size, follow-up duration, and the potential for residual confounding. A formal certainty-of-evidence assessment, such as GRADE, was not performed.

## Results

### 3.1 Study selection

A total of 150 records were identified through database searching. After removal of duplicates, 135 records remained and were screened based on titles and abstracts. Of these, 85 records were excluded due to irrelevance to the study objectives.

A total of 50 full-text articles were assessed for eligibility. Following detailed evaluation, 25 studies were excluded for the following reasons: failure to meet inclusion criteria (*n* = 7), lack of access to full text (*n* = 13), and publication in a language other than English (*n* = 5).

Ultimately, 25 studies met the predefined eligibility criteria and were included in the qualitative synthesis. The study selection process is summarized in the PRISMA flow diagram (Figure 1).

### 3.2 Risk of bias and methodological limitations

Risk of bias was assessed at the study level using ROBINS-I for non-randomized studies and RoB 2 for randomized controlled trials. Complete domain-level judgments and overall assessments are presented in Supplementary Tables S2A and S2B.

Among the 11 non-randomized studies included in the contemporary evidence synthesis, 5 studies (45.5%) were judged to have moderate overall risk of bias and 6 studies (54.5%) had serious overall risk of bias. No non-randomized study was judged to have low overall risk. The most frequent concern was bias due to confounding, reflecting non-random treatment allocation, differences in baseline operative risk, surgeon- and center-dependent treatment selection, and incomplete control of factors such as atrial fibrillation duration, left atrial size, mitral valve pathology, surgical approach, and left atrial appendage management.

Bias in outcome measurement was also an important concern. Rhythm surveillance varied from scheduled electrocardiography to Holter monitoring, pacemaker interrogation, and implantable loop recording. In several studies, prolonged monitoring was incomplete or declined over time, potentially leading to underdetection of asymptomatic atrial arrhythmia recurrence. Missing follow-up data were particularly important in studies reporting outcomes beyond 5–10 years.

The randomized trial by Gillinov et al. was judged to raise some concerns overall, primarily because rhythm outcome data were unavailable for a proportion of randomized participants. Randomization procedures and objective rhythm assessment using 3-day Holter monitoring were otherwise considered to have low risk of bias.

Overall, the evidence base was limited by residual confounding, treatment-selection bias, heterogeneous rhythm monitoring, and variable left atrial appendage management. These limitations are particularly relevant when interpreting reported associations with stroke, survival, and other long-term clinical endpoints. Because no formal certainty-of-evidence framework such as GRADE was applied, no overall certainty rating was assigned.

### 3.3 Early surgical ablation studies in mitral valve disease

Early studies established the Cox-Maze III procedure as an effective surgical strategy for the treatment of atrial fibrillation in patients undergoing mitral valve surgery (Table 1) Initial clinical experience demonstrated that concomitant Maze procedures could be safely performed without a significant increase in perioperative morbidity or mortality. Prospective cohort data reported restoration of sinus rhythm in approximately 90% of patients with chronic atrial fibrillation associated with mitral valve disease (14). Similarly, comparative analyses demonstrated significantly higher rates of sinus rhythm maintenance in patients undergoing mitral valve repair combined with the Cox-Maze procedure compared with valve surgery alone, along with a reduction in thromboembolic events (15, 47).

**Table 1:** Early surgical ablation studies and randomized evidence in mitral valve disease.

Study	Year	Study design	Population	Intervention	Follow-up	Key findings
Isobe et al. (14)	1998	Prospective cohort	30 patients with chronic AF + MV disease	Cox-Maze III+mitral surgery	∼2.1 years	∼90% sinus rhythm restoration; low mortality; low pacemaker rate
Handa et al. (15)	1999	Retrospective comparative	97 patients (Maze+repair vs repair alone)	Cox-Maze III+mitral repair	2 years	Higher sinus rhythm (74% vs 27%); reduced stroke risk
Kim et al. (47)	1999	Cohort study	Rheumatic MV disease + AF	Cox-Maze III + valve surgery	Not reported	High sinus rhythm conversion; acceptable perioperative risk
Ad et al. (16)	2002	Prospective cohort	53 patients	Cox-Maze III + mitral surgery	∼2.8 years	98% arrhythmia control; no stroke/TIA; pacemaker 19%
Deneke et al. (19)	2002	Prospective randomized trial	30 patients with chronic AF + MV disease	MVR + cooled-tip RFA Maze vs MVR alone	12 months	Higher sinus rhythm with adjunctive RFA Maze than MVR alone (80.0% vs 26.7%); no 30-day mortality
De Lima et al. (17)	2004	RCT	30 patients with permanent AF + MV disease	PVI vs Maze vs surgery alone	∼24 months	Maze and PVI superior to surgery alone for sinus rhythm
Doukas et al. (20)	2005	Randomized double-blind trial	97 patients with continuous AF undergoing MV surgery	MVS + left atrial RFA vs MVS alone	12 months	Higher sinus rhythm with RFA than control (44.4% vs 4.5%); similar complications and mortality
Albrecht et al. (18)	2009	RCT	60 patients	Cox-Maze III vs SPVI vs control	Long-term	Maze and SPVI equally effective; both reduced AF recurrence
Chevalier et al. (21)	2009	Prospective randomized multicenter trial	43 patients with persistent AF + MV disease	MVS + left atrial RFA vs MVS alone	12 months	Higher rhythm success with RFA than control; sinus rhythm at 12 months 95.2% vs 33.3%; similar stroke/complication rates
Wu et al. (22)	2010	Comparative cohort	60 patients with rheumatic MV disease + AF	MVR + Cox-Maze III	up to 10 years	∼79% freedom from AF at 10 years; durable outcomes

AF, atrial fibrillation; MV, mitral valve; MVR, mitral valve replacement; MVS, mitral valve surgery; RCT, randomized controlled trial; PVI, pulmonary vein isolation; SPVI, surgical pulmonary vein isolation; RFA, radiofrequency ablation; TIA, transient ischemic attack.

Subsequent studies confirmed the reproducibility of these findings. Cohort studies reported excellent long-term rhythm control, with arrhythmia elimination achieved in the majority of patients and no perioperative or late thromboembolic events (16). Randomized evidence further supported the efficacy of surgical ablation, demonstrating that both the Cox-Maze procedure and pulmonary vein isolation were superior to isolated mitral valve surgery for maintaining sinus rhythm (17). Additional randomized data showed comparable effectiveness between modified Cox-Maze III and pulmonary vein isolation, with both approaches significantly reducing atrial fibrillation recurrence (18).

Additional randomized studies further strengthened the evidence supporting concomitant surgical ablation during mitral valve surgery. A prospective randomized trial demonstrated that adding a modified Maze procedure using cooled-tip radiofrequency ablation during mitral valve replacement significantly improved sinus rhythm restoration compared with valve surgery alone (19). Similarly, a randomized double-blind trial reported superior maintenance of sinus rhythm after adjunctive left atrial radiofrequency ablation during mitral valve surgery without an increase in perioperative complications (20). These findings were further supported by a multicenter randomized trial, in which concomitant radiofrequency ablation achieved significantly higher rates of sinus rhythm maintenance than mitral valve surgery alone (21). Together, these studies provided important evidence supporting energy-based surgical ablation strategies in the early era of concomitant AF surgery.

Long-term observational data also demonstrated sustained efficacy of the Cox-Maze III procedure. In patients with rheumatic mitral valve disease, freedom from atrial fibrillation approached 80% at 10 years following concomitant mitral valve replacement and Maze surgery (22). Collectively, these early studies established the Cox-Maze III procedure as the reference standard for surgical treatment of atrial fibrillation in the setting of mitral valve disease, demonstrating high rates of sinus rhythm restoration, durable long-term outcomes, and acceptable safety profiles.

### 3.4 Cox-Maze IV and contemporary surgical ablation in mitral valve surgery

Contemporary evidence demonstrates that the Cox-Maze IV procedure provides effective and durable rhythm control in patients undergoing mitral valve surgery with concomitant atrial fibrillation (Table 2). Early prospective data reported freedom from atrial fibrillation of approximately 89% at 12 months, while also identifying key predictors of recurrence, including left atrial enlargement and incomplete lesion sets (23). These findings established the procedural efficacy and highlighted the importance of surgical technique in long-term outcomes.

**Table 2:** Cox-Maze IV and contemporary surgical ablation in mitral valve surgery.

Study	Year	Study design	Population	Intervention	LAA management	Follow-up	Rhythm monitoring/success definition	Key findings
Damiano et al. (23)	2011	Prospective cohort	282 patients with AF (mixed cardiac surgery; 66% concomitant procedures)	Cox-Maze IV	exclusion	12 months	ECG and Holter/prolonged monitoring; ATA recurrence defined as episode >30 s	Freedom from AF ∼89% at 1 year; predictors of recurrence: enlarged LA, incomplete posterior LA isolation, early atrial arrhythmias
Saint et al. (37)	2013	Cohort study	213 patients with MV disease and AF (MV alone vs MV + CM-IV)	Cox-Maze IV + MV surgery	Not reported	∼3 years	ECG/Holter follow-up; freedom from AF/ATA reported at 3, 6, and 12 months	No significant increase in operative mortality; CM-IV did not add procedural risk
Lawrance et al. (24)	2014	Retrospective cohort	356 patients (including MV cases)	Cox-Maze IV (RMT vs sternotomy)	excision/closure	2 years	ECG at follow-up visits + 24-h Holter/continuous monitoring at 3, 6, 12, and 24 months	
	Similar rhythm outcomes; ↓ morbidity and LOS with minimally invasive approach							
Gillinov et al. (29)	2015	RCT	260 patients with persistent AF undergoing MV surgery	Surgical ablation (PVI or biatrial Maze) vs no ablation	closure/occlusion	12 months	3-day Holter monitoring at 6 and 12 months; freedom from AF at both time points	
	Higher freedom from AF (63.2% vs 29.4%); ↑ pacemaker implantation							
Boano et al. (38)	2017	Cohort study	116 patients undergoing MV surgery (with vs without CM-IV cryoablation)	Cox-Maze IV (cryoablation)	suture closure	1 year	ECGs, 24–48 h Holter in selected patients, and pacemaker interrogation when applicable	
	Possible increase in postoperative heart failure; no clear difference in mortality							
Ad et al. (26)	2018	Prospective cohort	473 patients with MV surgery + AF	Cox-Maze (III/IV, predominantly IV)	amputation and closure	up to 7 years	HRS-based follow-up; mainly 24-h Holter; recurrence defined as atrial arrhythmia >30 s	SR: 90% (1 yr), 80% (5 yr), 66% (7 yr); stroke rate very low
Bogachev-Prokophiev et al. (31)	2021	Prospective cohort	242 patients with MV surgery + AF	Cox-Maze IV (cryo-based)	Exclusion	∼3–5 years	24-h Holter at 3, 6, and 12 months, then annually; recurrence >30 s	
	Freedom from AF: ∼79% (1 yr), ∼60% (5 yr); safe and reproducible							
Khiabani et al. (39)	2022	Prospective cohort	853 patients with AF (including ∼51% mitral valve surgery)	Cox-Maze IV (biatrial or left-sided lesion set)	closure	up to 10 years	ECG at visits + prolonged monitoring, including 24-h Holter, pacemaker interrogation, or loop recorder; recurrence >30 s	
	Freedom from atrial tachyarrhythmia: 92% (1 yr), 84% (5 yr), 77% (10 yr); predictors of recurrence included LA size, nonparoxysmal AF, and early postoperative arrhythmia							
Yates et al. (25)	2023	Propensity-matched cohort	377 patients (MV surgery + CM-IV)	Cox-Maze IV (RMT vs sternotomy)	excision/closure			
	up to 8 years	ECG + prolonged monitoring with Holter, pacemaker interrogation, or loop recorder; recurrence >30 s	Comparable long-term survival; better early rhythm with RMT					
Kim et al. (28)	2024	Multicenter propensity-matched cohort	2680 patients (AF + MV disease)	MV surgery + AF surgery vs MV alone	resection/obliteration	up to 15 years	12-lead ECG; Holter monitoring not performed in all patients	
	↓ mortality, ↓ stroke, ↓ MACCE; ↑ pacemaker risk							
Yi et al. (27)	2025	Cohort study	847 patients (MV ± CM-IV)	Cox-Maze IV	excision/oversewing/exclusion	up to 10 years	ECG at scheduled visits + prolonged monitoring using 24/72-h Holter, pacemaker interrogation, or loop recorder; recurrence >30 s	SR ∼80% (5 yr), ∼65% (10 yr); survival benefit vs no ablation
Ivert et al. (30)	2025	Registry cohort	∼700 patients (CM-IV + MV surgery vs MV alone)	Cox-Maze IV	closure	up to 8 years	Registry-based rhythm assessment; AF defined as episode ≥30 s on telemetry or 12-lead ECG	∼2 × higher pacemaker risk after CM-IV

AF, atrial fibrillation; SR, sinus rhythm; MV, mitral valve; MVR, mitral valve replacement; MVS, mitral valve surgery; PVI, pulmonary vein isolation; LA, left atrium/left atrial; LAA, left atrial appendage; RFA, radiofrequency ablation; MACCE, major adverse cardiac and cerebrovascular events; PPM, permanent pacemaker; ECG, electrocardiography; ATA, atrial tachyarrhythmia; NR, not reported.

Subsequent studies confirmed the reproducibility of these results across different surgical approaches. Retrospective analyses demonstrated that minimally invasive right mini-thoracotomy achieved comparable rhythm outcomes to sternotomy, with reduced morbidity and shorter hospital stay (24). Similarly, propensity-matched data showed improved early freedom from atrial tachyarrhythmia with minimally invasive approaches without compromising long-term survival (25).

Long-term observational data further support the durability of Cox-Maze IV. Cohort studies reported sustained sinus rhythm rates of approximately 90%, 80%, and 66% at 1, 5, and 7 years, respectively, with a very low incidence of thromboembolic events (26). These findings are consistent with more recent observational data demonstrating durable rhythm control and reporting an association between concomitant ablation and improved survival during follow-up of up to 10 years (27). A large multicenter propensity-matched cohort also reported lower mortality, stroke, and major adverse cardiac and cerebrovascular event rates among patients undergoing concomitant atrial fibrillation surgery (28). However, these non-randomized findings remain susceptible to residual confounding, treatment-selection bias, heterogeneity in patient characteristics, and differences in left atrial appendage management and therefore should not be interpreted as definitive evidence of causal benefit.

Randomized evidence also supports the use of surgical ablation in this setting. A landmark trial demonstrated significantly higher rates of freedom from atrial fibrillation at 12 months in patients receiving surgical ablation compared with mitral valve surgery alone, albeit with an increased incidence of permanent pacemaker implantation (29). This trade-off is further supported by registry data showing a higher long-term risk of pacemaker implantation following Cox-Maze IV in conjunction with mitral valve surgery (30).

Finally, alternative energy-based approaches, such as cryoablation, have shown comparable effectiveness within the Cox-Maze IV framework. Prospective cohort data demonstrated acceptable long-term freedom from atrial arrhythmia and a favorable safety profile (31). Collectively, these studies consistently support improved rhythm control with Cox-Maze IV and other contemporary surgical ablation strategies. Some observational studies also suggest possible improvements in survival and thromboembolic outcomes, although the magnitude and causal nature of these associations remain uncertain. An increased risk of permanent pacemaker implantation remains a consistent safety concern.

### 3.5 Lesion set configuration, energy sources, and surgical approach

The configuration of lesion sets is a critical determinant of the success of surgical ablation for atrial fibrillation in patients undergoing mitral valve surgery. Among the available strategies, biatrial lesion sets represent the most comprehensive approach, aiming to interrupt macro-reentrant circuits in both atria. This strategy has been associated with higher rates of sinus rhythm maintenance, particularly in patients with persistent or long-standing atrial fibrillation (32). However, the increased extent of ablation may be accompanied by a higher risk of conduction disturbances and subsequent permanent pacemaker implantation, reflecting a trade-off between procedural efficacy and safety (32, 33).

Comparative evidence indicates that more limited left atrial lesion sets may provide similar clinical outcomes in selected patient populations. Studies have demonstrated no significant differences in long-term survival, stroke, or freedom from atrial fibrillation between biatrial and left atrial ablation in patients undergoing mitral valve surgery, while more extensive ablation strategies may be associated with a higher incidence of postoperative complications (33). These findings suggest that routine use of biatrial ablation may not be necessary in all patients and that a tailored approach based on individual risk profiles is warranted.

The completeness of lesion sets appears to be a key determinant of procedural success. Incomplete ablation, particularly failure to achieve effective isolation of the posterior left atrium and pulmonary veins, has been consistently associated with arrhythmia recurrence (34, 35). This highlights that the continuity and transmurality of lesions may be more important than the overall extent of ablation. In this context, left atrial lesion sets incorporating posterior wall isolation remain a fundamental component of effective surgical strategies.

The choice of energy modality also influences procedural characteristics. Both radiofrequency ablation and cryoablation are widely used and have demonstrated comparable efficacy in achieving sinus rhythm maintenance. While cryoablation may be associated with more consistent lesion formation and favorable effects on early atrial remodeling, overall rhythm outcomes and safety profiles appear similar between techniques (36). As a result, the selection of energy source is often guided by anatomical considerations, lesion location, and surgeon experience rather than clear differences in long-term outcomes.

In addition to lesion configuration and energy delivery, the surgical approach may impact perioperative outcomes. Minimally invasive techniques, including right mini-thoracotomy, have been shown to achieve comparable rhythm control to conventional sternotomy while reducing surgical trauma and facilitating faster recovery (32, 34). These findings support the feasibility of less invasive approaches without compromising the effectiveness of concomitant atrial fibrillation ablation.

In summary, although biatrial lesion sets offer the most comprehensive ablation strategy, current evidence supports a more individualized approach. Procedural success depends not only on lesion extent but also on lesion completeness, energy modality, and patient-specific factors such as atrial size and disease duration. Tailoring the ablation strategy to the clinical context is therefore essential to optimize outcomes while minimizing procedural risk.

### 3.6 Safety and complications of concomitant surgical ablation

Although concomitant surgical ablation during mitral valve surgery has demonstrated clear benefits in rhythm control and long-term outcomes, it is also associated with specific procedural risks that must be carefully considered. Overall, the addition of surgical ablation does not appear to significantly increase operative mortality when performed in experienced centers, supporting its safety as an adjunct to mitral valve procedures (28, 37). However, the procedure is associated with increased operative complexity, including longer cardiopulmonary bypass and cross-clamp times.

One of the most consistently reported complications is the increased need for permanent pacemaker implantation. This risk is primarily attributed to injury or modification of the cardiac conduction system during extensive lesion creation, particularly with biatrial ablation strategies. Both randomized and large observational studies have demonstrated a higher incidence of pacemaker implantation in patients undergoing concomitant surgical ablation compared with mitral valve surgery alone (29, 30). This risk represents a key trade-off between improved rhythm control and potential conduction disturbances.

Perioperative complications such as bleeding, renal dysfunction, and prolonged hospitalization have also been reported, although these outcomes are generally influenced by patient comorbidities and surgical complexity rather than the ablation procedure itself. Evidence from studies of minimally invasive approaches suggests that less invasive techniques may reduce perioperative morbidity while maintaining comparable efficacy (24, 25).

Several observational studies reported lower long-term stroke or thromboembolic event rates among patients undergoing concomitant surgical ablation (27, 28). However, these findings should be interpreted cautiously. The included studies differed substantially in baseline patient characteristics, anticoagulation management, rhythm outcomes, surgical technique, and the use and method of left atrial appendage exclusion or occlusion. Because left atrial appendage management may independently influence thromboembolic risk, the observed associations cannot be attributed solely to surgical ablation or restoration of sinus rhythm. Residual confounding and treatment-selection bias also remain possible, particularly in retrospective and propensity-matched studies. Accordingly, the available evidence suggests a possible thromboembolic benefit but does not establish a definitive reduction in stroke caused by concomitant ablation.

Despite these benefits, certain complications remain procedure-specific. Cryoablation-based approaches have been associated in some studies with an increased risk of postoperative heart failure, although findings are not entirely consistent across studies (38). Additionally, early postoperative atrial arrhythmias are common and may predict late recurrence, underscoring the importance of careful postoperative monitoring and management (23).

In summary, concomitant surgical ablation during mitral valve surgery is consistently associated with improved rhythm control. However, it may increase procedural complexity and the risk of permanent pacemaker implantation. Possible associations with lower thromboembolic event rates and improved survival have been reported mainly in observational studies and remain uncertain because of residual confounding, differences in patient selection, and variable left atrial appendage management.

## Discussion

This systematic review provides an overview of concomitant surgical ablation in patients with atrial fibrillation undergoing mitral valve surgery, with particular attention to differences in procedural strategy and outcome assessment. The available evidence consistently supports better sinus rhythm restoration and maintenance following concomitant ablation, particularly with contemporary Cox-Maze IV-based approaches (28, 39). In contrast, evidence regarding stroke, survival, and other major clinical endpoints is less certain. Most data for these outcomes originate from observational cohorts and propensity-matched analyses that remain vulnerable to residual confounding, treatment-selection bias, heterogeneity in patient characteristics, and variation in concomitant left atrial appendage management.

The results of this review highlight that procedural success is influenced not only by the use of ablation itself but also by the specific characteristics of the ablation strategy. Biatrial lesion sets remain the most comprehensive approach and are generally associated with superior rhythm outcomes, particularly in patients with persistent or long-standing atrial fibrillation. However, this increased efficacy must be balanced against a higher risk of conduction disturbances and the subsequent need for permanent pacemaker implantation. In contrast, more limited left atrial lesion sets may offer acceptable outcomes in selected patients, suggesting that a tailored approach based on individual patient characteristics is appropriate.

Another key finding is the importance of lesion completeness. Although lesion extent remains important, procedural success is also strongly influenced by lesion completeness and the underlying atrial substrate. Studies using endocardial mapping have demonstrated that achieving durable conduction block and fully transmural lesions may be more important than simply increasing the number of lesions (40, 41). Residual conduction gaps have been identified despite apparently successful ablation, highlighting the limitations of relying solely on lesion-set design. More recent investigations incorporating functional substrate mapping have further emphasized the role of atrial remodeling and fibrosis in determining long-term rhythm outcomes (42). These findings suggest that successful surgical ablation depends not only on the chosen lesion set but also on lesion durability and the characteristics of the underlying atrial substrate. Across multiple studies, incomplete ablation, particularly inadequate isolation of the posterior left atrium and pulmonary veins, has been associated with higher rates of arrhythmia recurrence (23, 35). This suggests that the quality and continuity of lesion sets may be more critical than their overall extent. Advances in energy delivery, including radiofrequency and cryoablation technologies, have facilitated the creation of more consistent transmural lesions, contributing to improved procedural outcomes. However, no clear superiority of one energy modality over another has been established, and the choice of technique is often guided by anatomical considerations and surgical expertise.

In addition to efficacy, safety remains a critical consideration. The available evidence indicates that concomitant surgical ablation does not appear to significantly increase operative mortality when performed in experienced centers (28, 37). Nevertheless, it is associated with increased procedural complexity and a higher incidence of permanent pacemaker implantation, particularly with more extensive lesion sets (29, 30). Perioperative complications may also be influenced by patient comorbidities, procedural complexity, and surgical approach.

Some included studies reported associations between concomitant surgical ablation and lower rates of stroke, mortality, or major adverse cardiac and cerebrovascular events (27, 28). These findings are clinically relevant but should be regarded as suggestive rather than definitive. Most supporting studies were observational, and patients selected for ablation may have differed from those undergoing mitral valve surgery alone in age, comorbidity burden, atrial fibrillation duration, atrial size, operative risk, center experience, and postoperative management. Although propensity-score methods may reduce measured baseline differences, they cannot eliminate residual confounding from unmeasured or incompletely measured variables.

Interpretation of thromboembolic outcomes is additionally complicated by variability in left atrial appendage management. Exclusion, occlusion, resection, or oversewing of the left atrial appendage was performed inconsistently across studies and may independently affect stroke risk, as supported by randomized evidence from the LAAOS III trial (43). Differences in anticoagulation strategies, rhythm-monitoring intensity, and maintenance of sinus rhythm further limit attribution of observed stroke reductions specifically to the ablation procedure.

Because the present review used a qualitative rather than meta-analytic design, it was not possible to generate pooled estimates, quantify statistical heterogeneity, or formally evaluate the robustness of these associations. The current evidence therefore supports a possible relationship between concomitant ablation and improved long-term clinical outcomes but does not establish that ablation independently reduces stroke or improves survival.

These findings have important clinical implications. Concomitant surgical ablation should be considered in eligible patients undergoing mitral valve surgery because of its consistently demonstrated benefits for rhythm control. Possible effects on stroke, survival, and other harder clinical endpoints may provide additional support for the procedure, but these outcomes remain less certain and should not be presented as established causal benefits. At the same time, individualized decision-making remains essential, taking into account factors such as atrial fibrillation type, duration of arrhythmia, left atrial size, and overall surgical risk.

Mitral valve pathology and operative strategy may also influence outcomes. Patients undergoing mitral repair and those undergoing mitral replacement may differ in disease severity, atrial remodeling, anticoagulation requirements, and long-term thromboembolic risk. Similarly, rheumatic mitral valve disease is often associated with more advanced atrial enlargement, fibrosis, and persistent atrial fibrillation than degenerative mitral disease, which may affect both rhythm success and clinical outcomes after ablation (27, 28). Therefore, comparisons across studies should account for differences in mitral repair vs. replacement and rheumatic vs. degenerative disease, although these distinctions were not consistently reported in the included literature.

Concomitant left atrial reduction or remodeling may also influence rhythm outcomes, particularly in patients with marked left atrial enlargement. Recent evidence suggests that left atrial volume reduction combined with mitral valve surgery and AF ablation may improve rhythm outcomes compared with ablation alone, although the effect has generally been reported as a trend rather than definitive superiority (10, 44). This is clinically relevant because enlarged left atrial size is a recognized predictor of AF recurrence after surgical ablation. However, reporting of atrial reduction techniques was inconsistent across the studies included in this review, precluding formal assessment of its independent effect on long-term rhythm outcomes. The increasing use of minimally invasive approaches further supports the feasibility of integrating ablation into contemporary surgical practice without compromising patient recovery. Emerging robotic-assisted and robot-enhanced hybrid approaches may represent the next stage in the evolution of minimally invasive AF surgery. Recent reports have described robotic epicardial ablation as the surgical component of staged hybrid strategies that combine minimally invasive surgical lesion creation with subsequent electrophysiological mapping and catheter-based completion of residual lesions (45, 46). Although evidence remains limited and these approaches were not represented in the studies included in the present review, they may further improve procedural precision and expand treatment options for selected patients with complex atrial fibrillation.

Despite these advances, several limitations of the current evidence base should be acknowledged. The majority of included studies are observational in design, which introduces potential bias and limits causal inference. Considerable heterogeneity exists in patient populations, lesion sets, energy modalities, and follow-up protocols, complicating direct comparison across studies. A major methodological limitation is the variability in rhythm-monitoring protocols and definitions of treatment success. Studies differed in the use of intermittent ECGs, Holter monitoring, scheduled rhythm assessments, and prolonged or continuous monitoring, each with different sensitivities for detecting recurrent atrial arrhythmias. Definitions of recurrence also varied with respect to blanking periods, antiarrhythmic drug use, monitoring intensity, and episode duration thresholds. As demonstrated by studies comparing intermittent and continuous monitoring after surgical ablation, recurrence rates may be underestimated when less intensive surveillance strategies are used (23). Additional limitations include restriction to English-language publications, lack of protocol registration, and absence of quantitative synthesis. Importantly, 13 potentially eligible articles could not be retrieved in full text and were therefore excluded. This may have reduced the comprehensiveness of the evidence base, introduced availability bias, and potentially influenced both the magnitude and direction of the review's conclusions if the unavailable studies differed systematically from the included literature. Finally, long-term data beyond 10–15 years remain relatively limited.

Future research should focus on well-designed multicenter randomized trials comparing different lesion sets and energy modalities, as well as on the development of standardized definitions for rhythm success and recurrence. Further investigation into patient selection criteria and the role of minimally invasive and hybrid approaches is also warranted. A more individualized approach to surgical ablation, guided by patient-specific characteristics and disease complexity, is likely to represent the next stage in optimizing outcomes for patients with atrial fibrillation undergoing mitral valve surgery.

## Conclusion

Concomitant surgical ablation during mitral valve surgery is consistently associated with improved restoration and maintenance of sinus rhythm in patients with atrial fibrillation. The Cox-Maze IV procedure represents the contemporary standard, offering effective rhythm control with reduced technical complexity compared with earlier cut-and-sew techniques. However, surgical ablation may increase procedural complexity and the risk of permanent pacemaker implantation, particularly with more extensive lesion sets. Some observational studies suggest possible reductions in thromboembolic events and possible survival benefit, but these associations remain uncertain because of residual confounding, heterogeneous patient selection, variable left atrial appendage management, and the absence of quantitative synthesis. The choice of lesion set, energy modality, and surgical approach should therefore be individualized according to atrial fibrillation type, atrial substrate, left atrial size, comorbidities, and operative risk. Overall, the strongest and most consistent evidence supports a benefit in rhythm outcomes, whereas effects on stroke, survival, and other harder clinical endpoints remain less certain.

## Data Availability

The original contributions presented in the study are included in the article/Supplementary Material, further inquiries can be directed to the corresponding author.
