# Supplementary material for: Concomitant surgical ablation for atrial fibrillation in mitral valve surgery: a systematic review of clinical outcomes and surgical strategies
Source: Front Cardiovasc Med. 2026 Aug 13;13:1857413. doi: 10.3389/fcvm.2026.1857413 (PMC13519401; doi:10.3389/fcvm.2026.1857413)
Supplement: Supplementary file 1 [file Supplementaryfile1.docx]

**Supplementary Materials**

***Supplementary Table S1.*** *Detailed database-specific search strategy*

| **Database** | **Search strategy** |
| --- | --- |
| PubMed | ("atrial fibrillation"[Title/Abstract] OR "Atrial Fibrillation"[MeSH Terms] OR AF[Title/Abstract]) AND ("mitral valve surgery"[Title/Abstract] OR "mitral valve repair"[Title/Abstract] OR "mitral valve replacement"[Title/Abstract] OR "mitral valve"[Title/Abstract]) AND ("surgical ablation"[Title/Abstract] OR "Maze"[Title/Abstract] OR "Cox-Maze"[Title/Abstract] OR "radiofrequency ablation"[Title/Abstract] OR cryoablation[Title/Abstract] OR "concomitant ablation"[Title/Abstract]) AND ("1991/01/01"[Date - Publication] : "2026/01/31"[Date - Publication]) |
| Embase | ('atrial fibrillation'/exp OR 'atrial fibrillation':ti,ab OR af:ti,ab) AND ('mitral valve surgery':ti,ab OR 'mitral valve repair':ti,ab OR 'mitral valve replacement':ti,ab OR 'mitral valve':ti,ab) AND ('surgical ablation':ti,ab OR maze:ti,ab OR 'cox-maze':ti,ab OR 'radiofrequency ablation':ti,ab OR cryoablation:ti,ab OR 'concomitant ablation':ti,ab) AND [1991-2026]/py |
| Cochrane Library | ("atrial fibrillation" OR AF) AND ("mitral valve surgery" OR "mitral valve repair" OR "mitral valve replacement") AND ("surgical ablation" OR Maze OR Cox-Maze OR "radiofrequency ablation" OR cryoablation) |
| Web of Science | TS=("atrial fibrillation" OR AF) AND TS=("mitral valve surgery" OR "mitral valve repair" OR "mitral valve replacement") AND TS=("surgical ablation" OR Maze OR Cox-Maze OR "radiofrequency ablation" OR cryoablation OR "concomitant ablation"); Timespan: 1991–2026 |

Supplementary Table S2A. ROBINS-I assessment of non-randomized studies

Judgments: L = Low risk; M = Moderate risk; S = Serious risk; C = Critical risk; NI = No information.

| **Study** | **D1 Confounding** | **D2 Selection of participants** | **D3 Classification of interventions** | **D4 Deviations from intended interventions** | **D5 Missing data** | **D6 Measurement of outcomes** | **D7 Selection of reported results** | **Overall** |
| --- | --- | --- | --- | --- | --- | --- | --- | --- |
| Damiano et al. [24], 2011 | M | L | L | L | L | M | M | M |
| Saint et al. [33], 2013 | S | M | L | L | L | M | M | S |
| Lawrance et al. [25], 2014 | S | M | L | L | M | M | M | S |
| Boano et al. [34], 2017 | S | M | L | L | L | L | M | S |
| Ad et al. [27], 2018 | M | M | L | L | M | M | M | M |
| Bogachev-Prokophiev et al. [32], 2022 | M | M | L | L | M | M | M | M |
| Khiabani et al. [35], 2022 | M | M | L | L | S | S | M | S |
| Yates et al. [26], 2023 | S | M | L | L | M | S | M | S |
| Kim et al. [29], 2024 | M | M | L | L | M | L | M | M |
| Yi et al. [28], 2025 | S | M | L | L | M | M | M | S |
| Ivert et al. [31], 2025 | M | M | L | L | L | L | M | M |

Supplementary Table S2B. RoB 2 assessment of the randomized trial

Judgments: L = Low risk; SC = Some concerns; H = High risk.

| **Study** | **D1 Randomization process** | **D2 Deviations from intended interventions** | **D3 Missing outcome data** | **D4 Measurement of outcome** | **D5 Selection of reported result** | **Overall** |
| --- | --- | --- | --- | --- | --- | --- |
| Gillinov et al. [30], 2015 | L | L | SC | L | L | SC |
